# Supplementary material for: Electrochemical biosensors in healthcare services: bibliometric analysis and recent developments
Source: PeerJ. 2023 Jun 27;11:e15566. doi: 10.7717/peerj.15566 (PMC10312160; doi:10.7717/peerj.15566)
Supplement: Supplemental Information 1 [file peerj-11-15566-s001.docx]

**Supplementary Table 1.** Ten most popular terms, along with their frequency

| **Keyword** | **Frequency** | **Total link strength** |
| --- | --- | --- |
| Electrochemical biosensor | 2472 | 18072 |
| Biosensor | 1084 | 8352 |
| Sensor | 909 | 7328 |
| Nanoparticles | 787 | 6520 |
| Gold nanoparticles | 651 | 5754 |
| DNA | 535 | 4041 |
| Electrode | 520 | 4138 |
| Immobilization | 422 | 3608 |
| Graphene | 391 | 3276 |
| Biosensors | 374 | 2879 |
