# Supplementary figures and images for: Electrochemical biosensors in healthcare services: bibliometric analysis and recent developments
Source: PeerJ. 2023 Jun 27;11:e15566. doi: 10.7717/peerj.15566 (PMC10312160; doi:10.7717/peerj.15566)

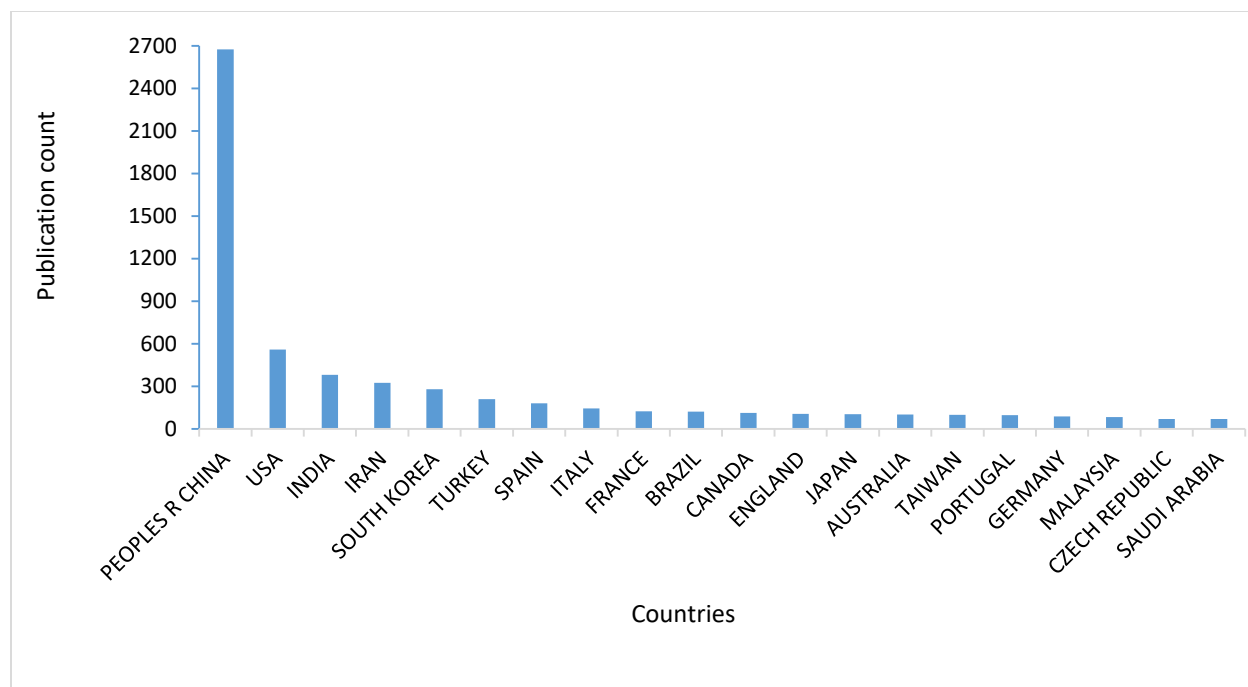

Supplementary Figure 2. Best 20 countries by number of publications

Supplement: Supplemental Information 3 [file peerj-11-15566-s003.pdf]

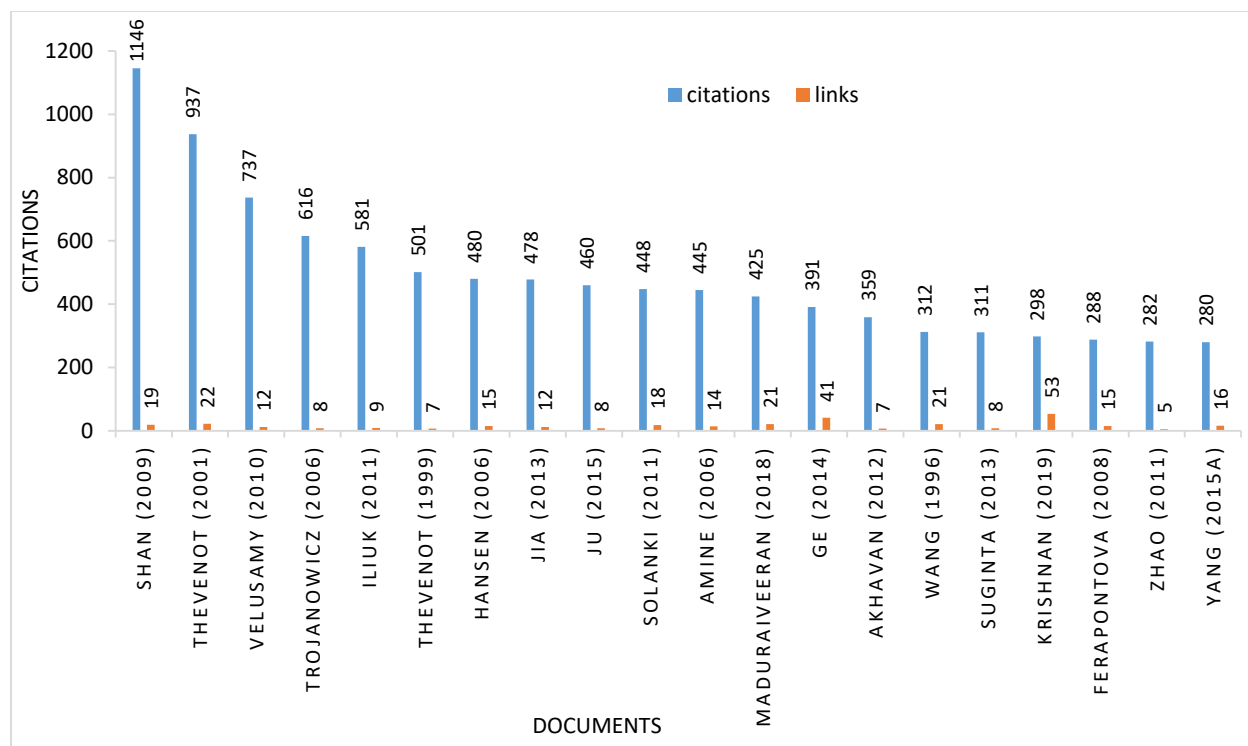

Supplementary Figure 4. Top 20 documents with their citations and links

Supplement: Supplemental Information 5 [file peerj-11-15566-s005.pdf]
