# Supplementary material for: Electrochemical biosensors in healthcare services: bibliometric analysis and recent developments
Source: PeerJ. 2023 Jun 27;11:e15566. doi: 10.7717/peerj.15566 (PMC10312160; doi:10.7717/peerj.15566)
Supplement: Supplemental Information 6 [file peerj-11-15566-s006.pdf]

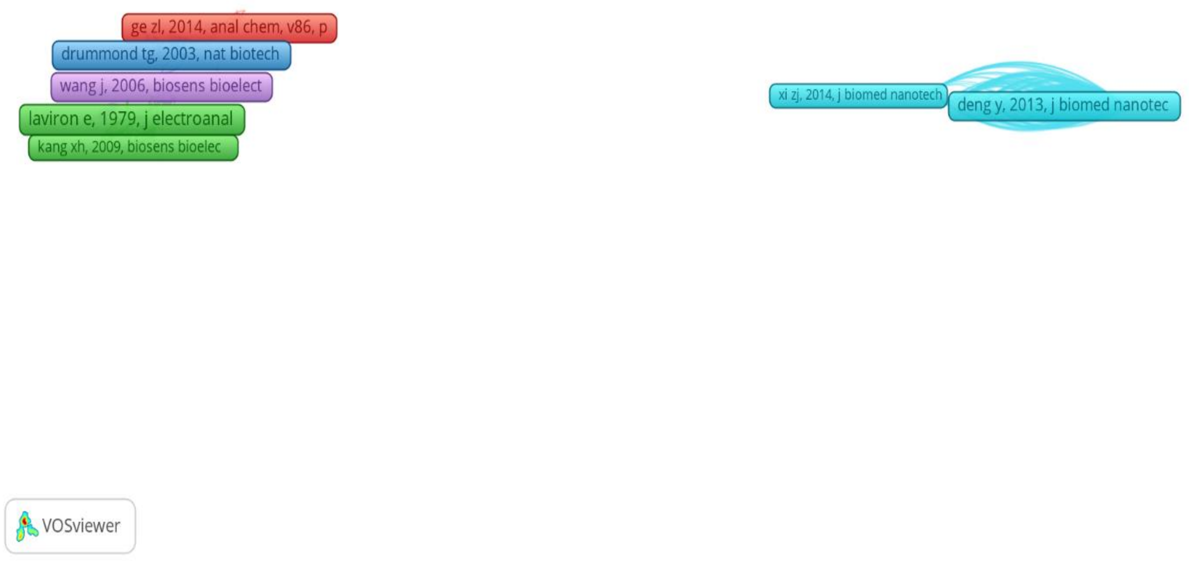

Supplementary Figure 5. Co-citation analyses of reference. Colors indicate cluster of related terms.
